# Epidemiologic, Phenotypic, and Structural Characterization of Aminoglycoside-Resistance Gene *aac(3)-IV*

**DOI:** 10.3390/ijms21176133

**Published:** 2020-08-25

**Authors:** Michel Plattner, Marina Gysin, Klara Haldimann, Katja Becker, Sven N. Hobbie

**Affiliations:** Institute of Medical Microbiology, University of Zurich, 8006 Zurich, Switzerland; mplattner@imm.uzh.ch (M.P.); mgysin@imm.uzh.ch (M.G.); khaldimann@imm.uzh.ch (K.H.); kbecker@imm.uzh.ch (K.B.)

**Keywords:** antimicrobial resistance, aminoglycosides, apramycin, acetyltransferase, *aac(3)-IV*

## Abstract

Aminoglycoside antibiotics are powerful bactericidal therapeutics that are often used in the treatment of critical Gram-negative systemic infections. The emergence and global spread of antibiotic resistance, however, has compromised the clinical utility of aminoglycosides to an extent similar to that found for all other antibiotic-drug classes. Apramycin, a drug candidate currently in clinical development, was suggested as a next-generation aminoglycoside antibiotic with minimal cross-resistance to all other standard-of-care aminoglycosides. Here, we analyzed 591,140 pathogen genomes deposited in the NCBI National Database of Antibiotic Resistant Organisms (NDARO) for annotations of apramycin-resistance genes, and compared them to the genotypic prevalence of carbapenem resistance and 16S-rRNA methyltransferase (RMTase) genes. The 3-*N*-acetyltransferase gene *aac(3)-IV* was found to be the only apramycin-resistance gene of clinical relevance, at an average prevalence of 0.7%, which was four-fold lower than that of RMTase genes. In the important subpopulation of carbapenemase-positive isolates, *aac(3)-IV* was nine-fold less prevalent than RMTase genes. The phenotypic profiling of selected clinical isolates and recombinant strains expressing the *aac(3)-IV* gene confirmed resistance to not only apramycin, but also gentamicin, tobramycin, and paromomycin. Probing the structure–activity relationship of such substrate promiscuity by site-directed mutagenesis of the aminoglycoside-binding pocket in the acetyltransferase AAC(3)-IV revealed the molecular contacts to His124, Glu185, and Asp187 to be equally critical in binding to apramycin and gentamicin, whereas Asp67 was found to be a discriminating contact. Our findings suggest that aminoglycoside cross-resistance to apramycin in clinical isolates is limited to the substrate promiscuity of a single gene, rendering apramycin best-in-class for the coverage of carbapenem- and aminoglycoside-resistant bacterial infections.

## 1. Introduction

Decreasing antibiotic efficacy in the treatment of drug-resistant bacterial infections is one of the greatest global health threats of our times, prompting the WHO to prioritize bacterial pathogens and define clinical needs for the development of new antibiotics [1]. With the bacterial cell wall and ribosome being the two most successful therapeutic targets in antibacterial history, it is not surprising that major pharmacological advancements have arisen from improving existing drug classes to evade resistance mechanisms, confer improved activity, and reduce toxicity. A major benefit of such an approach lies in the pre-existing clinical validation of both the molecular target and the drug class, and a proven clinical utility that facilitates early adoption by treatment guidelines, and thus, market penetration [2].

One such success story is that of aminoglycosides, a drug class comprising potent antimicrobial agents with broad-spectrum activity that bind to the bacterial ribosome and inhibit bacterial protein-synthesis [3].

However, the efficacy of many clinically established aminoglycosides eroded over time due to the emergence and spread of aminoglycoside resistance. Such resistance was mainly caused by genetic vectors coding for aminoglycoside-modifying enzymes that inactivate the drug by acetylation, phosphorylation, or nucleotidylation [4]. The introduction of amikacin, arbekacin, and plazomicin quite successfully overcame some of the resistance mechanisms that compromised the efficacy of gentamicin and tobramycin. However, all three were still affected by at least some of the clinically relevant resistance mechanisms, such as aminoglycoside acetyltransferases *aac(6′)*, *aac(2′)*, *aph(2″)*, and the 16S-rRNA methyltransferases [5,6]. Further chemical modification and scaffold evolution of aminoglycoside antibiotics are mandatory to restore the clinical utility of this drug class, which has long been listed as essential medicines by the WHO [7,8].

Apramycin was proposed as a possible next-generation aminoglycoside, and it is currently the only new aminoglycoside in clinical development [9,10]. Apramycin is a monosubstituted 2-deoxystreptamine with an unusual bicyclic octose moiety on C4, providing significant structural differentiation from the 4,6-disubstituted deoxystreptamines gentamicin, tobramycin, amikacin, arbekacin, and plazomicin, which currently define the standard of care in treating infections with an aminoglycoside antibiotic. This structural distinction was demonstrated to intrinsically evade almost all aminoglycoside-resistance mechanisms of clinical relevance, including the m^7^G1405 methylation of bacterial 16S rRNA, a mechanism that was termed pan-aminoglycoside resistance because it inactivates the whole range of the 4,6-disubstituted deoxystreptamines [11].

Few resistance mechanisms have been described that protect bacterial pathogens from apramycin. Previous reports have shown that apramycin is rendered ineffective by *aac(3)-IV*, a resistance gene encoding the aminoglycoside 3-*N*-acetyltransferase IV (AAC(3)-IV), an enzyme that exhibits a broad substrate range including not only gentamicin and tobramycin, but also apramycin [12]. Other mechanisms reported in the literature have remained more elusive and less well-documented, including acetyltransferase *apmA* [13], a putative *aac(1)* gene [14], and two m^1^A1408 16S rRNA methyltransferases, *npmA* [15] and *kamB* [16].

In the present study, we aimed to assess the potential clinical relevance of apramycin resistance genes. We analyzed the NCBI National Data Base of Antibiotic Resistance Organisms (NDARO) to study the occurrence of resistance genes of confirmed or putative relevance for apramycin susceptibility. We then compared the prevalence of the *aac(3)-IV* resistance gene in the most clinically relevant group of carbapenem-resistant clinical isolates with that of 16S-rRNA methyltransferase genes. We further elucidated the phenotypic resistance profile conferred by the *aac(3)-IV* gene in clinical isolates and in engineered *Escherichia coli* strains heterologously expressing *aac(3)-IV*. Lastly, we probed the structure–function relationship of AAC(3)-IV substrate promiscuity at the molecular level by site-directed mutagenesis of the substrate-binding site.

## 2. Results

### 2.1. Genotypic Epidemiology of Apramycin Resistance

First, we wanted to understand the potential clinical relevance of pre-existing apramycin resistance. Towards this end, we studied the resistance-gene annotations of clinical isolates deposited in the NDARO.

Few genes have been reported in the scientific literature to be associated with apramycin resistance, with varying levels of experiment evidence: *aac(3)-IV*, *apmA*, *npmA*, *kamB*, and *aac(1)*. We previously challenged the existence of a true *aac(1)* gene, because alignment of the amino acid sequences revealed putative *aac(1)* genes to have very high sequence homology to *aac(3)* genes [11]. Of the 7010 entries in the NDARO list of reference genes, 689 entries were associated with aminoglycoside resistance, and six were identified as being associated with apramycin resistance: *aac(3)-IV* (also annotated as *aac(3)-IVa*), *apmA* (two variants), *npmA*, and *kamB* (two variants). In comparison, seventeen entries were identified as m^7^G1405 16S rRNA methyltransferases (RMTases): *armA*, *rmtA*, *rmtB*, *rmtC*, *rmtD*, *rmtE*, *rmtF*, *rmtG*, *rmtH*, *grm*, and *sgm*. The prevalence of RMTase genes was selected as a reference value for the prevalence of apramycin-resistance genes because methylation of the drug-binding pocket by an RMTase results in high-level resistance to all clinically relevant aminoglycosides, but retains susceptibility to apramycin [11].

**Figure 1:**
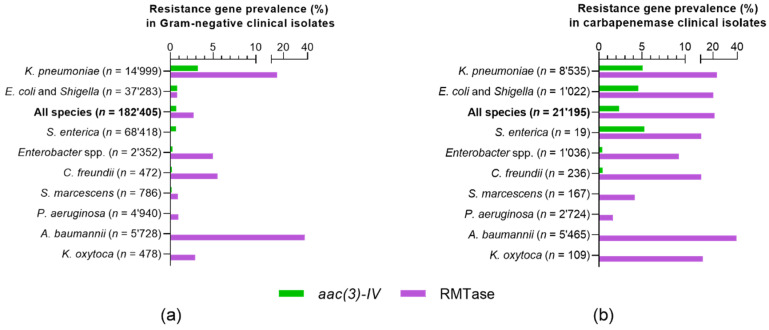
Genomic prevalence of the *aac(3)-IV* gene in Gram-negative clinical isolates in comparison to RMTase resistance genes. (**a**) Gene-annotation analysis of 182,405 clinical-isolate genomes deposited in NCBI National Database of Antibiotic Resistant Organisms (NDARO) on 24 June 2020. (**b**) Gene-annotation analysis in a subpopulation of 21,195 carbapenemase-positive (CP) clinical isolates. Gene *aac(3)-IV* confers resistance to gentamicin, tobramycin, and apramycin, among others. RMTases confer pan-aminoglycoside resistance to all 4,6-disubstituted deoxystreptamines comprising all standard-of-care aminoglycoside antibiotics, but remain susceptible to apramycin.

An analysis of the gene annotations for the 182,405 clinical-isolate genomes deposited in the NDARO database revealed 1269 isolates (0.7%) with an apramycin-resistance gene, compared to 4941 isolates (2.7%) with an RMTase gene (Figure 1A and Table S1). Virtually all isolates indicated for potential resistance to apramycin were due to the annotation of the *aac(3)-IV* gene. Genes *apmA* and *npmA* annotated to only two isolates each (0.001%), namely, to gastrointestinal pathogens *Campylobacter jejuni* and *Clostridium difficile*, respectively (Table S1). No annotation was found for the *kamB* gene.

Since we were interested in assessing the specific performance of apramycin in pathogen populations of high clinical relevance and medical need, we further defined a subpopulation of clinical isolates with carbapenemase-gene annotations to find 21,195 (11.6%) of clinical-isolate genomes in the database being carbapenemase-positive (CP). Of these, 495 (2.3%) were found to also be positive for *aac(3)-IV*, compared to 4551 (21.5%) CP isolates that were positive for an RMTase gene (Figure 1B and Table S2). No other apramycin-resistance gene besides *aac(3)-IV* was annotated to the 21,195 clinical CP isolates. In this study, we therefore focused on *aac(3)-IV* as the only apramycin-resistance gene of clinical relevance.

The genomic prevalence of the *aac(3)-IV* and RMTase genes varied considerably between individual bacterial species. For *Klebsiella pneumoniae* isolates, for instance, higher rates of both *aac(3)-IV* and RMTase genes were found than for most other species. For *Acinetobacter baumannii,* the rate of RMTase genes was highest, but *aac(3)-IV* gene annotation resulted in only a single hit overall (<0.001%), and no hits in the CP population. The prevalence of *aac(3)-IV* was generally low when compared to that of RMTase genes (Figure 1 and Tables S1 and S2). The *aac(3)-IV* gene was detected only in Gram-negative bacterial isolates, and not in Gram-positive isolates.

### 2.2. Phenotypic Characterization of aac(3)-IV

Next, we studied the phenotype of the *aac(3)-IV* gene by broth-microdilution antimicrobial susceptibility testing to determine the minimal inhibitory concentrations of selected aminoglycosides comprising 4,6-disubstituted 2-deoxystreptamines (DOS), 4,5-disubstituted DOS, and the monosubstituted DOS apramycin. To avoid overlapping phenotypic effects by the presence of other aminoglycoside-resistance genes, we selected clinical isolates with a positive genome annotation for *aac(3)-IV*, but negative for any other aminoglycoside-resistance gene. We could identify only three *E. coli* isolates that matched these criteria: AG173, AG380, and AG381 (Table S3).

The three *E. coli aac(3)-IV* isolates were resistant to apramycin, as expected, and showed varying degrees of nonsusceptibility to gentamicin, tobramycin, sisomicin, netilmicin, and paromomycin (Table 1). All three isolates were consistently susceptible to amikacin and plazomicin.

The phenotypic variation in minimal-inhibitory-concentration (MIC) values between the three clinical isolates, the lack of a sufficiently large sample number of *aac(3)-IV* isolates to determine MIC distribution, and the possibility of unknown interfering effects in clinical isolates such as membrane permeability prompted us to engineer a small panel of isogenic *E. coli* strains recombinantly expressing *aac(3)-IV*. The aminoglycoside-susceptibility profile of the recombinant strains resembled that of the clinical isolates, and MICs increased with promoter strength, as was expected (Table 1).

### 2.3. Structural Analysis of Aminoglycoside Binding to AAC(3)-IV

Since *aac(3)-IV*, unlike all other *aac(3)* subtypes, conferred phenotypic resistance to aminoglycosides of three structurally distinct subclasses, we decided to study the structural basis for the substrate promiscuity of aminoglycoside acetyltransferase AAC(3)-IV. To better understand the potential molecular interactions between AAC(3)-IV and bound aminoglycosides, we analyzed the macromolecular structure of apo AAC(3)-IV (PDB ID: 6MN3) and AAC(3)-IV in complex with apramycin (PDB ID: 6MN4) and gentamicin (PDB ID: 6MN5).

**Figure 2:**
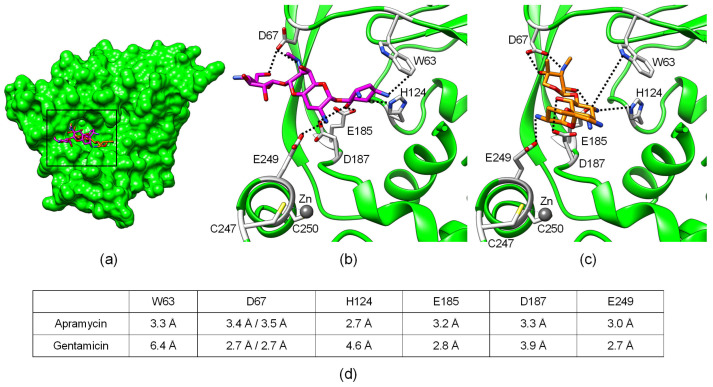
Crystal structure of AAC(3)-IV in complex with apramycin (PDB ID: 6MN4) or gentamicin (PDB ID: 6MN3). (**a**) Structural overview of full protein showing superimposition of bound substrates apramycin (magenta) and gentamicin (orange). Detailed view of (**b**) apramycin and (**c**) gentamicin in the binding pocket. Amino acids modified in this study are highlighted in gray. Residues D67, D187, and E249 are differently rotated in the apramycin versus the gentamicin structure. (**d**) Shortest intermolecular distances between amino acid side chains and ligand, corresponding to dashed lines in (**b**,**c**).

Analysis of the substrate-binding pocket revealed four negatively-charged carboxylic side chains of aspartate and glutamate residues that pointed towards the bound ligand, with intermolecular distances close to that of hydrogen bonds (Figure 2). The side chains of Asp67, Asp187, and Glu249 appeared to be rotamers of different orientations in the apramycin versus the gentamicin model, wheras the side chain of Glu185 had the same orientation in both models (Figure 2B,C).

**Figure 3:**
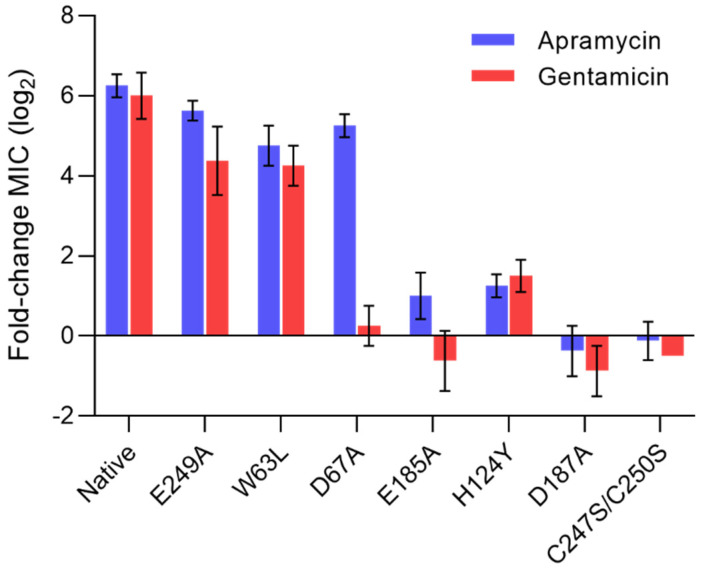
Functional assessment of native and mutant protein AAC(3)-IV. Phenotypic susceptibility to apramycin and gentamicin in recombinant *E. coli* strains displayed as fold-changes in minimal-inhibitory-concentration (MIC) relative to a susceptible wild-type strain lacking *aac(3)-IV* (mean ± SE; *n* = 4).

To probe whether these four interactions contribute to ligand promiscuity, we mutated Asp67, Glu185, Asp187, and Glu249 individually to alanine. Mutation Glu249Ala resulted in slightly lower MICs when compared to native protein, but seemed to not be detrimental to overall enzyme activity (Figure 3). The two mutations Glu185Ala and Asp187Ala abolished phenotypic resistance to both apramycin and gentamicin, highlighting a critical role of the two residues in the binding of both apramycin and gentamicin. In contrast, the Asp67Ala mutation only abolished resistance to gentamicin, but not to apramycin, suggesting a discriminating role despite similar molecular proximity to both drugs being suggested by the structural models.

We further mutated Trp63 to leucine to probe the importance of a possible hydrophobic interaction between the aminoglycoside and tryptophan. The molecular interaction with Trp63 seemed to also be equally important to the binding of both substrates, even though the Trp63Leu mutation resulted in only a moderate decrease of MICs of both drugs, suggesting that it makes less critical contributions to the binding of apramycin and gentamicin. The His124Tyr mutant was generated to probe whether replacing histidine with a similarly bulky residue with an alcohol group affected ligand preferences, as suggested by a previous finding [17]. The His124Tyr mutation restored susceptibility to both drugs, suggesting this molecular contact was equally critical in binding both apramycin and gentamicin.

A structural analysis of the substrate-binding pocket further highlighted the proximity of a zinc ion, which might be expected to contribute to correct protein folding and structural protein integrity (Figure 2). To probe the importance of the zinc ion, we mutated the two cysteines, i.e., 247 and 250, to serine, rendering AAC(3)-IV inactive, as judged by full drug susceptibility of the mutant, similar to that of the wild-type strain (Figure 3).

## 3. Discussion

The medical need for new antibacterial therapeutics has mainly arisen due to the emergence and spread of antimicrobial resistance to well-established existing therapies. One of the main objectives of developing new antibiotics must therefore be to evade existing resistance mechanisms in the target pathogen population.

Apramycin has been described by several independent research groups as an aminoglycoside antibiotic that evades almost all aminoglycoside-resistance mechanisms typically encountered in clinical settings, and consequently demonstrated phenotypic coverage of multidrug-resistant pathogens superior to that of other aminoglycosides [9,11,18,19,20,21,22,23,24,25,26,27]. It was, therefore, surprising that one study reported an *aac(3)-IV* annotation in 9 out of 98 (9.2%) *Enterobacterales* isolates in a preselected panel of drug-resistant human clinical isolates [28]. This was contrasted by other antimicrobial-susceptibility studies that found much smaller rates of apramycin resistance in large study populations, including one study from our own research group that looked at over a thousand multidrug-resistant clinical isolates [11,29].

Such phenotypic studies are often compromised by limited sample size and potentially biased sample selection, making it more difficult to draw general epidemiologic conclusions. Therefore, we decided to complement the various phenotypic studies reported in the literature with the present genotypic study that utilized a large genomic database of drug-resistant bacteria with over half a million of entries to broaden our understanding of the prevalence of apramycin resistance. Such an approach is limited to annotations of known resistance genes, and it infers phenotypic resistance on the basis of genomic information. However, unlike phenotypic antimicrobial-susceptibility testing, it provided an opportunity to analyze unprecedented large numbers of bacterial clinical isolates, and therefore, to deliver the most comprehensive epidemiologic assessment of *aac(3)-IV* and RMTases to date.

On the basis of the hypothetical assumption that the absence of resistance-genes in the annotation is associated with particular phenotypic susceptibility, we concluded that >99% of all studied clinical isolates and 98% of the CP subpopulation are genotypically susceptible to apramycin. In contrast, susceptibility to standard-of-care aminoglycosides was lower, at <97% and <79%, respectively, when based on RMTase gene annotations alone and not taking into account the high number of aminoglycoside-modifying enzymes that inactivate the various standard-of-care aminoglycosides but not apramycin. The higher levels of aminoglycoside resistance in CP isolates might not be surprising, given the frequent co-occurrence of different resistance genes, including that of RMTases with carbapenemases [6,30,31,32]. Of note, we did not detect any *aac(3)-IV* genes in the critical pathogen populations of carbapenem-resistant *A. baumannii* and *Pseudomonas aeruginosa*. This may suggest that *A. baumannii* infections, which tend to be notoriously difficult to treat and are often classified as pan-aminoglycoside resistant due to the high prevalence of RMTases, would remain susceptible to apramycin treatment. Indeed, this genotypic observation corresponds well to previous phenotypic studies with *A. baumannii* clinical isolates [11,20,26].

Other apramycin-resistance genes of epidemiologic relevance were not found in the NDARO. Acetyltransferase gene *apmA* seemed to be of very low prevalence in Gram-negative pathogens. The only two gene annotations in Gram-negative genomes were found in 2 out of 9681 *C. jejuni* isolates. The gene was initially described in methicillin-resistant *Staphylococcus aureus* (MRSA) isolated from bovine and porcine isolates, and has since been found in other Gram-positives, too [13]. Gene annotations for *npmA,* an m^1^A1408 rRNA methyltransferase, were similarly rare, and only found in 2 out of 2319 *C. difficile* isolates. Both *C. jejuni* and *C. difficile* are gastrointestinal pathogens not typically treated with aminoglycosides, but with other medications such as azithromycin or vancomycin [33,34].

Antimicrobial-susceptibility testing of clinical isolates and recombinant strains confirmed that *aac(3)-IV* confers resistance to not only apramycin, but also to other, structurally different aminoglycosides. This result seemingly contrasted one of our previous studies where a synthetic *aac(3)-IV* gene conferred resistance to apramycin, but not to gentamicin or tobramycin [11]. We only later found that the underlying reference gene accession number X01385, rooting back to its first description in 1984 [35], does contain a single nucleotide insertion that results in a frameshift and changes the amino acid sequence of the C-terminal end of AAC(3)-IV (Figure S1). We further noticed that the *aac(3)-IV* reference genes used in genome annotations (e.g., accession number WP_001199192.1 in the NDARO) with a length of 258 amino acids fall short of the 267 amino acids of the *aac(3)-IV* open reading frame (e.g., NCBI reference sequence WP_000093041.1). We cloned both variants with and without the nine amino acid N-terminal leader sequence (MSSAVECNVV), but found no difference in their antibiotic-resistance profiles (Table S4).

AAC(3)-IV did not seem to inactivate amikacin and plazomicin. Both aminoglycosides carry a (*S*)-4-amino-2-hydroxybutyrate (AHBA) modification at the 1-*N* position of the 2-DOS ring. The low affinity of amikacin to AAC(3)-IV was previously described using an enzymatic in-vitro assay [36]. Modeling of the active site suggested that the AHBA modification sterically interfered with proper positioning of the aminoglycoside in the active cleft (Figure S2). Interestingly, the smaller 1-*N* ethyl modification in netilmicin, which sets netilmicin apart from sisomicin, did not seem to protect the molecule from acetylation by AAC(3)-IV.

The finding that MIC values for the recombinant strains increased with promoter strength provides a possible explanation for the phenotypic variability in clinical isolates by transcriptional regulation. However, direct comparison between recombinant strains and clinical isolates has limitations. Other non-specific off-target effects, for instance variations in drug uptake and in membrane permeability, may likewise contribute to MIC variability, and will need to be elucidated in future studies using larger panels of resistant bacterial isolates.

In order to assess the contribution of specific amino acid residues to substrate binding, we compared the MICs of apramycin and gentamicin between native proteins and proteins with site-specific mutations in the aminoglycoside-binding site. Mutagenesis of His124, Glu185, or Asp187 resulted in full drug susceptibility, underscoring the functional importance of these three residues in substrate binding. Residue Asp67 appeared to provide the only molecular interaction that discriminated between apramycin and gentamicin. Mutagenesis to Asp67Ala resulted in susceptibility to gentamicin, but retained resistance to apramycin. This suggests a critical role of the molecular interaction with Asp67 in the binding of gentamicin, but not of apramycin, despite the similar intramolecular distances observed for both substrates. Our interpretation is that molecular interaction with residues His124, Glu185, and Asp187 is key to substrate binding and promiscuity, but that additional contact to Asp67 is needed to provide sufficient affinity for gentamicin binding.

Molecular interactions with Glu249 and Trp63 seem to also contribute to substrate binding, albeit to a minor extent. Disrupting the interaction between the carboxyl group of Glu249 and the 2′-amino groups of apramycin or gentamicin, respectively, had only a minor effect on the resistance phenotype of *aac(3)-IV*. The contribution of Trp63 to substrate binding may be that of a hydrophobic interaction, but Trp63 might also play a role in properly positioning the ligand in relation to acetyl coenzyme A (Figure S2).

We conclude that a network of strong molecular interactions with carboxylic rotamer side chains provides the space and flexibility in the substrate binding pocket to accommodate structurally diverse aminoglycosides. It appears to be this unique substrate promiscuity that renders subtype IV the only member of the AAC(3) family that confers cross-resistance to apramycin. The clinical prevalence of *aac(3)-IV*, however, appears to be relatively low, and other apramycin resistance mechanisms can be considered to be of low clinical relevance in pathogen populations targeted by aminoglycoside therapy. Collectively, and in comparison to other aminoglycoside antibiotics, apramycin appears to provide best-in-class coverage of multidrug-resistant Gram-negative pathogens including *A baumannii*, *P. aeruginosa*, and *Enterobacterales*.

## 4. Materials and Methods

### 4.1. Genomic Database Mining

The gene-annotation metafile of the NCBI National Database of Antibiotic Resistance Organisms (NDARO) was downloaded on 24 June 2020. It contained 591,140 entries of resistant bacterial isolates and a catalog of 7058 resistance genes. Gene annotations were screened for genes previously hypothesized or confirmed to confer apramycin resistance: *aac(3)-IVa*, *apmA*, *npmA*, and *kamB*. The metafile was further screened for annotations of 16S-rRNA (guanine(1405)-N(7))-methyltransferase (RMTase) and of carbapenem-resistance genes. The list of RMTase genes used in the search comprised *armA*, *rmtA*, *rmtB*, *rmtC*, *rmtD*, *rmtE*, *rmtF, rmtG, and rmtH*. The list of carbapenem-resistance genes used in the search comprised gene families *bla2, blaAFM, blaAIM, blaALG11, blaALG6, blaALI, blaANA, blaAXC, blaB, blaBPEDO, blaBIC, blaBJP, blaBKC, blaCAM, blaCAR, blaCAU, blaCPS, blaCRD, blaCRH, blaCRP, blaCVI, blaDHT2, blaDIM, blaEAM, blaEBR, blaECM, blaECV, blaEFM, blaELM, blaESP, blaEVM, blaFEZ, blaFIA, blaFIM, blaFPH, blaFRI, blaGIM, blaGOB, blaGPC, blaGRD, blaHMB, blaIMI, blaIMP, blaIND, blaJOHN, blaKHM, blaKPC, blaL1, blaMOC, blaMSI, blaMUS, blaMYO, blaMYX, blaNDM, blaORR, blaOXA-10, OXA-23, OXA-24, OXA-48, OXA-51, OXA-55, OXA-58, OXA-60, OXA-62, OXA-134, OXA-143, OXA-211, OXA-212, OXA-213, OXA-214, OXA-215, OXA-229, OXA-372, blaPFM, blaPLN, blaPNGM, blaPOM, blaPST, blaRSA2, blaRm3, blaSFC, blaSFH, blaSHN, blaSME, blaSPG, blaSPM, blaSPN79, blaSPR, blaSPS, blaSTA, blaTHIN-B, blaTMB, blaTRU, blaTTU, blaTUS, blaVCC, blaVIM, blaZOG, cfiA, cphA, cphA1*, and *varG*. All resistance-gene annotation names are based on the NDARO reference gene catalog; where applicable, the annotation search included all gene variants of the aforementioned gene families (e.g., *blaB-1* to *blaB-39*). The list of carbapenem-resistance genes further included those genes of the *blaGES* family reported to hydrolyze carbapenems (*blaGES* gene subtypes 2, 4, 5, 6, 14, 15, 16, 18, 20, 21, and 24), as well as individual genes *blaADC-68* and *blaSHV-38*. Bacterial isolates with annotation hits were sorted according to species and assigned to genotypic clusters, inferring a putative phenotype from the presence or absence of resistance genes.

### 4.2. Bacterial Strains

All strains used in this study are summarized in Table S3. Bacterial clinical isolates deposited in the University of Zurich repository were screened for resistance-gene annotations to identify three isolates with a gene annotation for only *aac(3)-IV*, i.e., no other aminoglycoside-resistance gene that might interfere with the phenotypic interpretation of *aac(3)-IV*. A set of *E. coli* strains recombinantly expressing *aac(3)-IV* under defined constitutive promoter control was engineered as previously described [11]. In brief, a synthetic *aac(3)-IV* gene was cloned into a low-copy number plasmid with pBR322 origin of replication, an ampicillin-resistance cassette, and a T7 terminator sequence. Gene expression was controlled by an insulated constitutive promotor of defined strength. The −10 hexamers *gtatct* (+), *taggct* (++), and *taatat* (+++) were chosen to provide a range of promoter strengths increasing by about half-log increments, defined as relative promoter units (RPUs) 0.009, 0.030, and 0.119 in the original publication [37]. Mutant genes were constructed by PCR mutagenesis (Agilent Quick Change Lightning).

### 4.3. Antimicrobial-Susceptibility Testing

Minimal inhibitory concentrations (MICs) were determined by broth microdilution according to CLSI standards and as described previously [11]. In brief, the bacterial inoculum was prepared from fresh overnight agar plates, and the inoculated assay plates were incubated at 35 ± 2 °C for 16 to 20 h prior to readout.

### 4.4. Structural Analysis

The PDB models and corresponding density maps for AAC(3)-IV apo structure (PDB ID: 6MN3), AAC(3)-IV apramycin complex (PDB ID: 6MN4), and AAC(3)-IV gentamicin complex (PDB ID: 6MN5) were downloaded from RCSB PDB [38]. The AAC(3)-IV active site model was generated, and protein-to-ligand distances were measured with UCSF Chimera [39]. The His154 rotamer was modeled according to the published AAC(3)-IIIb model [40]. Acetyl-co-enzyme-A was modeled according to the published AAC(3)-VIa model [41].

## Figures and Tables

**Table 1:** Aminoglycoside susceptibility of *E. coli aac(3)-IV*.

	*E. coli* Clinical *aac(3)-IV* Isolates				*E. coli* Recombinant *aac(3)-IV* Strains			
	ATCC	AG173	AG380	AG381		Promoter Strength		
Aminoglycoside	25,922				WT	+	++	+++
Apramycin	4	256–512	>512	>512	2	128	>512	>512
Gentamicin	0.5–1	8–16	32–64	64–128	0.25	8	64	128–256
Tobramycin	0.5–1	32	64–128	128	0.5	16	128–256	256–512
Amikacin	2	1	2–4	4	0.5–1	0.25–0.5	0.25	0.25–0.5
Sisomicin	0.5	8–16	16	32	0.125–0.25	2	16–32	128
Netilmicin	0.5	8–16	32–64	32	0.125–0.25	4–8	32–64	128
Plazomicin	0.5–1	0.5–1	1	1–2	0.25–0.5	0.125	0.125–0.25	0.125
Paromomycin	4	8	8	16	1	2	16	64–128

## Supplementary Materials

The following are available online at https://www.mdpi.com/1422-0067/21/17/6133/s1. Table S1: Prevalence of apramycin- vs. RMTase resistance gene annotations in human clinical isolates, Table S2: Prevalence of apramycin- vs. RMTase resistance gene annotations in the carbapenemase-positive subpopulation of human clinical isolates, Table S3: Strains used in this study, Table S4: Aminoglycoside susceptibility of recombinant *E. coli* strains constitutively expressing N-terminally truncated wild-type or mutant *aac(3)-IV* under defined promoter control, Figure S1: Amino acid sequence alignment of the native wild-type AAC(3)-IV with the NDARO reference sequence and the reference sequence originally reported in the literature, Figure S2: AAC(3)-IV activity model.
