# Supplementary material for: Epidemiologic, Phenotypic, and Structural Characterization of Aminoglycoside-Resistance Gene aac(3)-IV
Source: Int J Mol Sci. 2020 Aug 25;21(17):6133. doi: 10.3390/ijms21176133 (PMC7504452; doi:10.3390/ijms21176133)
Supplement: Supplementary file 1 [file ijms-21-06133-s001.pdf]

International Journal of *Molecular Sciences*

MDPI

Table S1. Prevalence of apramycin- vs. RMTase resistance gene annotations in human clinical isolates deposited in the NCBI National Database of Antibiotic Resistant Organisms (NDARO) as of June 24, 2020.

	**Clinical Isolate Resistance Gene Annotations**										
	**Total**	***aac(3)-IV***		***apmA***		***npmA***		***kamB***		***RMTase***	
	n	*n*	%	*n*	%	*n*	%	*n*	%	*n*	%
**All genomes**	182,405	1,265	0.69%	2	0.00%	2	0.00%	0	0.00%	4,941	2.71%
**Gram-negatives**											
A. baumannii	5,728	1	0.02%	0	0.00%	0	0.00%	0	0.00%	2,150	37.53%
P. aeruginosa	4,940	3	0.06%	0	0.00%	0	0.00%	0	0.00%	47	0.95%
E. coli / Shigella	37,283	298	0.80%	0	0.00%	0	0.00%	0	0.00%	300	0.80%
K. pneumoniae	14,999	485	3.23%	0	0.00%	0	0.00%	0	0.00%	2,250	15.00%
K. oxytoca	478	0	0.00%	0	0.00%	0	0.00%	0	0.00%	14	2.93%
Enterobacter spp.	2,352	6	0.26%	0	0.00%	0	0.00%	0	0.00%	117	4.97%
S. marcescens	786	1	0.13%	0	0.00%	0	0.00%	0	0.00%	7	0.89%
C. freundii	472	1	0.21%	0	0.00%	0	0.00%	0	0.00%	26	5.51%
M. morganii	47	2	4.26%	0	0.00%	0	0.00%	0	0.00%	3	6.38%
S. enterica	68,418	451	0.66%	0	0.00%	0	0.00%	0	0.00%	11	0.02%
C. jejuni	9,681	0	0.00%	2	0.02%	0	0.00%	0	0.00%	0	0.00%
**Gram-positives**											
C. difficile	2,319	0	0.00%	0	0.00%	2	0.09%	0	0.00%	0	0.00%
E. faecium	7,215	0	0.00%	0	0.00%	0	0.00%	0	0.00%	0	0.00%

Table S2. Prevalence of apramycin- vs. RMTase resistance gene annotations in the carbapenemase-positive subpopulation of human clinical isolates deposited in the NCBI National Database of Antibiotic Resistant Organisms (NDARO) as of June 24, 2020.

	**Carbapenemase-Positive Clinical Isolate Resistance Gene Annotations**										
	**Total**	***aac(3)-IV***		***apmA***		***npmA***		***kamB***		***RMTase***	
	n	*n*	%	*n*	%	*n*	%	*n*	%	*n*	%
**All CP genomes**	21,195	495	2.34%	0	0.00%	0	0.00%	0	0.00%	4,551	21.47%
**Gram-negatives**											
A. baumannii	5,465	0	0.00%	0	0.00%	0	0.00%	0	0.00%	2,145	39.25%
P. aeruginosa	2,724	1	0.04%	0	0.00%	0	0.00%	0	0.00%	44	1.62%
E. coli / Shigella	1,022	47	4.60%	0	0.00%	0	0.00%	0	0.00%	210	20.55%
K. pneumoniae	8,535	433	5.07%	0	0.00%	0	0.00%	0	0.00%	1,992	23.34%
K. oxytoca	109	0	0.00%	0	0.00%	0	0.00%	0	0.00%	13	11.93%
Enterobacter spp.	1,036	4	0.39%	0	0.00%	0	0.00%	0	0.00%	96	9.27%
S. marcescens	167	0	0.00%	0	0.00%	0	0.00%	0	0.00%	7	4.19%
C. freundii	236	1	0.42%	0	0.00%	0	0.00%	0	0.00%	25	10.59%
M. morganii	13	1	7.69%	0	0.00%	0	0.00%	0	0.00%	3	23.08%
S. enterica	19	1	5.26%	0	0.00%	0	0.00%	0	0.00%	2	10.53%
C. jejuni	618	0	0.00%	0	0.00%	0	0.00%	0	0.00%	0	0.00%
**Gram-positives**											
C. difficile	0	0	n/a	0	n/a	0	n/a	0	n/a	0	n/a
E. faecium	1	0	0.00%	0	0.00%	0	0.00%	0	0.00%	0	0.00%

Table S3. Strains used in this study.

Strain	Aminoglycoside Resistance Gene	Gene Variation	Promoter Strength[a]
Clinical isolates			
ATCC 25922	none	-	-
AG173	*aac(3)-IV*	None (native)	-
AG380	*aac(3)-IV*	None (native)	-
AG381	*aac(3)-IV*	None (native)	-
Recombinant			
DH5α	none	-	-
EC118	*aac(3)-IV*	None (native)	+
EC111	*aac(3)-IV*	None (native)	++
EC119	*aac(3)-IV*	None (native)	+++
EC116	*aac(3)-IV*	Truncated[b]	+
EC110	*aac(3)-IV*	Truncated[b]	++
EC117	*aac(3)-IV*	Truncated[b]	+++
EC109	*aac(3)-IV*	H154A	++
EC274	*aac(3)-IV*	H124Y	+
EC275	*aac(3)-IV*	D67A	+
EC276	*aac(3)-IV*	E185A	+
EC277	*aac(3)-IV*	D187A	+
EC278	*aac(3)-IV*	E249A	+
EC279	*aac(3)-IV*	E248A E249A	+
EC280	*aac(3)-IV*	C247A C250A	+
EC281	*aac(3)-IV*	C247S C250S	+
EC282	*aac(3)-IV*	W63A	+
EC283	*aac(3)-IV*	W63L	+

[a]Insulated constitutive promoters as described by [37]; [b]N-terminal truncation by 9 amino acids corresponding to the reference gene (see Figure S1).

Table S4. Aminoglycoside susceptibility of recombinant *E. coli* strains constitutively expressing N-terminally truncated wild-type or mutant *aac(3)-IV* under defined promoter control.

	**WT**	**WT**	**WT**	**H154A**
Strain	**EC116**	**EC110**	**EC117**	**EC109**
Promoter	**+**	**++**	**+++**	**++**
9-aa leader	**no**	**no**	**no**	**no**
MIC (mg/L)				
Apramycin	128-256	>512	>512	1-2
Gentamicin	16	64	128-256	0-125-0.25
Tobramycin	32	128	512	0.125
Sisomicin	4	32	128	0.125
Netilmicin	16-32	32-64	128	0.125-0.25
Paromomycin	4-8	16	64-128	0.5
Amikacin	0.5	0.5	0.25-0.5	0.5
Plazomicin	0.125	0.25	0.25	0.125

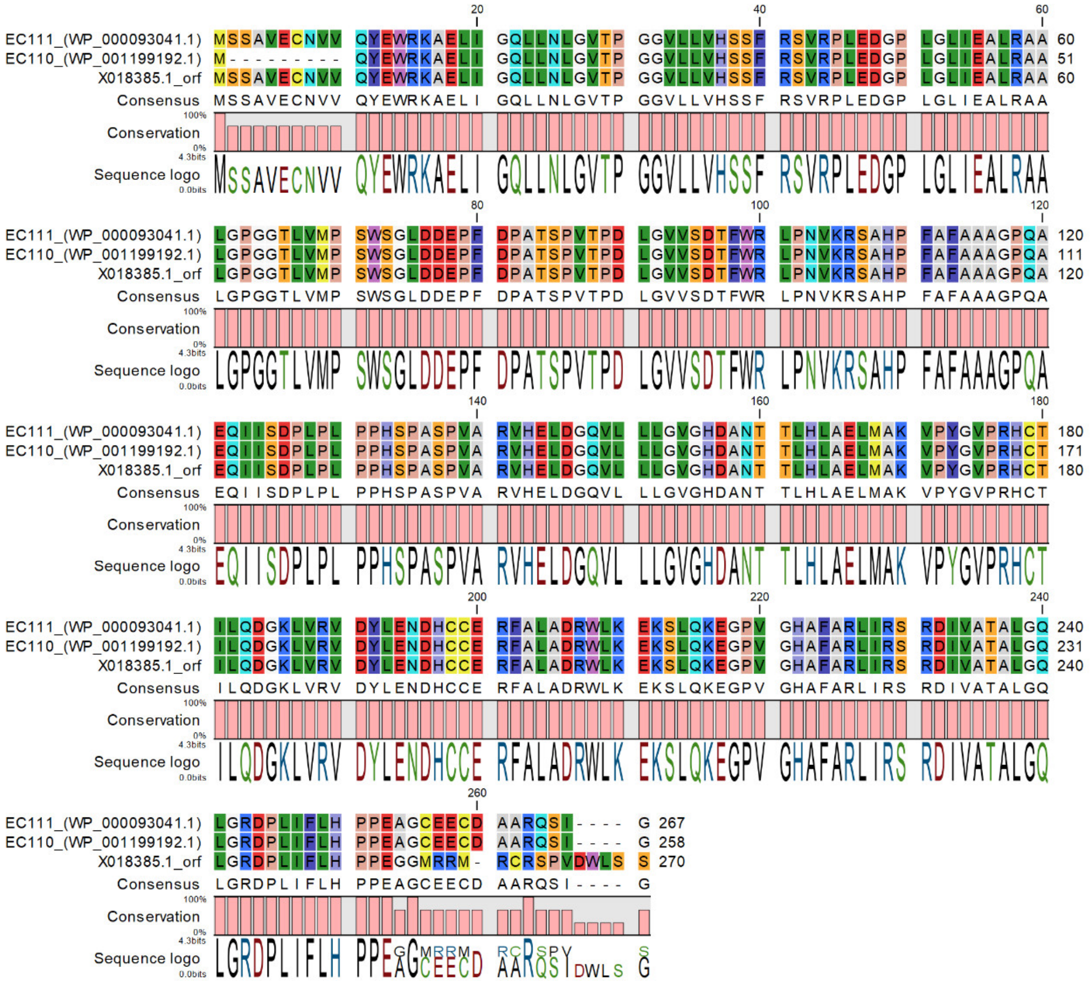

Figure S1. Amino acid sequence alignment of the native wild-type AAC(3)-IV (WP_000093041, e.g. EC111) with the NDARO reference sequence (WP_001199192, e.g. EC110), and the reference sequence originally reported in the literature (nucleic acid sequence X01385; *Mol. Gen. Genet. 1984, 193:179-187*). The NDARO reference gene *aac(3)-IV* used in gene annotations lacks the first nine amino acids SSAVECNVV that are present in the native protein. The nucleic acid sequence with gene accession number X01385 contains a single-nucleotide insertion mutation. This frameshift results in a C-terminal amino acid sequence of GGMRRMRCRSPVDWLSS instead of the native AGCEECDAARQSIG. It is conceivable to assume the replacement of the two cysteines held in place by a zinc atom may destabilize the tertiary structure of the protein and thus affect substrate promiscuity. The phenotype of such frameshift mutant was previously described to confer resistance to apramycin but not gentamicin or tobramycin, lacking the substrate promiscuity intrinsic for native AAC(3)-IV (*J. Antimicrob. Chemother. 2019, 74:944-952*).

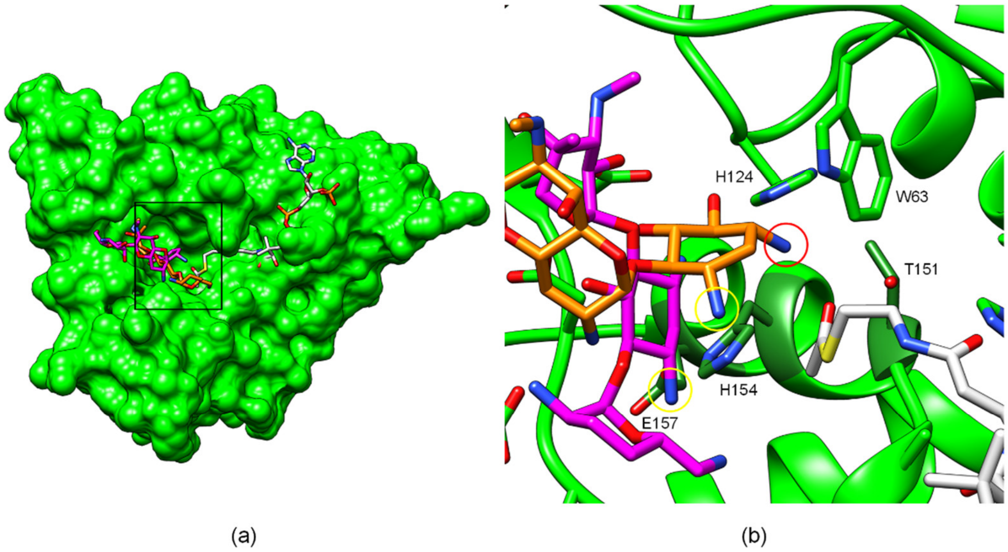

Figure S2. AAC(3)-IV activity model showing apramycin (magenta), gentamicin (orange) and acetyl-co-enzyme-A (light grey). This model is based on the published AAC(3)-IV* H154A models (PDB ID: 6MN3, 6MN4 and 6MN5). The histidine 154 rotamer rotation was modeled based on a AAC(3)-IIIb model (PDB accession: 6mb9) and the acetyl-co-enzyme-A was modeled according to a AAC(3)-VI model (PDB ID: 6BC4). (A) general overview. (B) Magnified view of the active site. The supposed catalytic triade (T151, H154 and E157) is colored in dark green (based on ref [40]). The 3-amino groups of apramycin and gentamicin are indicated by yellow circles. Modification of the 1-amino-group (indicated by a red circle) most likely results in steric hindrance that prevents proper positioning of the 3-amino group for acetylation, drug susceptibility is therefore maintained as observed for 1-*N*-modified amikacin and plazomicin in this study.
